# Supplementary material for: Metabolomic Profiling of Cerebral Palsy Brain Tissue Reveals Novel Central Biomarkers and Biochemical Pathways Associated with the Disease: A Pilot Study
Source: Metabolites. 2019 Feb 2;9(2):27. doi: 10.3390/metabo9020027 (PMC6409919; doi:10.3390/metabo9020027)
Supplement: Supplementary file 1 [file metabolites-09-00027-s001.pdf]

metabolites

MDPI

Article

Metabolomic Profiling of Cerebral Palsy Brain Tissue Reveals Novel Central Biomarkers and Biochemical Pathways Associated with the Disease: A Pilot Study

Zeynep Alpay Savasan [1,2,*], Ali Yilmaz [3], Zafer Ugur [3], Buket Aydas [4], Ray O. Bahado-Singh[1,2], and Stewart F. Graham [2,3]

1 Department of Obstetrics and Gynecology, Maternal Fetal Medicine Division, Beaumont Health System, 3811 W. 13 Mile Road, Royal Oak, MI 48073, USA; Ray.Bahado-Singh@beaumont.org
2 Oakland University-William Beaumont School of Medicine, Beaumont Health, 3811 W. 13 Mile Road, Royal Oak, MI 48073, USA; Stewart.Graham@beaumont.org
3 Beaumont Research Institute, Beaumont Health, 3811 W. 13 Mile Road, Royal Oak, MI 48073, USA; Ali.Yilmaz@beaumont.org (A.Y.); Zafer.Ugur@beaumont.org (Z.U.)
4 Departments of Mathematics and Computer Sciences, Albion College, 611 E. Porter St., Albion, MI 49224, USA; baydas@albion.edu (B.A.)

* Correspondence: Zeynep.AlpaySavasan@beaumont.org; Tel: +1-248-712-4595

Received: 9 January 2019; Accepted: 31 January 2019; Published: date

Abstract: Cerebral palsy (CP) is one of the most common causes of motor disability in childhood, with complex and heterogeneous etiopathophysiology and clinical presentation. Understanding the metabolic processes associated with the disease may aid in the discovery of preventive measures and therapy. Tissue samples (caudate nucleus) were obtained from post-mortem CP cases (n = 9) and age- and gender-matched control subjects (n = 11). We employed a targeted metabolomics approach using both ^{1}H NMR and direct injection liquid chromatography-tandem mass spectrometry (DI/LC-MS/MS). We accurately identified and quantified 55 metabolites using ^{1}H NMR and 186 using DI/LC-MS/MS. Among the 222 detected metabolites, 27 showed significant concentration changes between CP cases and controls. Glycerophospholipids and urea were the most commonly selected metabolites used to develop predictive models capable of discriminating between CP and controls. Metabolomics enrichment analysis identified folate, propanoate, and androgen/estrogen metabolism as the top three significantly perturbed pathways. We report for the first time the metabolomic profiling of post-mortem brain tissue from patients who died from cerebral palsy. These findings could help to further investigate the complex etiopathophysiology of CP while identifying predictive, central biomarkers of CP.

Keywords: cerebral palsy; metabolomics; ^{1}H NMR; targeted mass spectrometry; metabolic pathways

PACS: J0101

HMDB	Compound ID	Mean (SD) of Control (uM)	Mean (SD) of CP (uM)	p-value	q-value (FDR)	Fold Change
HMDB00294	Urea	59.236 (37.499)	184.144 (14.774)	0.0074 (W)	0.299	-3.11
HMDB00148	L-Glutamic acid	499.627 (15.680)	6.767 (13.764)	0.0106 (W)	0.299	73.83
HMDB13456	PC(o-22:2(13Z,16Z)/22:3(10Z,13Z,16Z))	1.187 (0.902)	0.335 (0.379)	0.0125 (W)	0.299	3.54
HMDB08276	PC(20:0/20:2(11Z,14Z))	0.265 (0.190)	0.051 (0.110)	0.0166 (W)	0.299	5.16
HMDB13450	PC(o-22:0/22:6(4Z,7Z,10Z,13Z,16Z,19Z))	0.847 (0.710)	0.231 (0.404)	0.0166 (W)	0.299	3.66
HMDB00195	Inosine	8.082 (4.627)	14.333 (6.338)	0.0201	0.299	-1.77
HMDB13333	3-Hydroxy-9-hexadecenoylcarnitine	0.061 (0.062)	0.129 (0.076)	0.0204 (W)	0.299	-2.13
HMDB10379	LysoPC(14:0)	5.237 (1.153)	4.151 (0.665)	0.0224	0.299	1.26
HMDB13433	PC(o-18:1(9Z)/22:0)	1.334 (0.714)	0.638 (0.487)	0.023	0.299	2.09
HMDB13453	PC(o-22:1(13Z)/22:3(10Z,13Z,16Z))	0.281 (0.180)	0.133 (0.069)	0.0248	0.299	2.12
HMDB07991	PC(16:0/22:6(4Z,7Z,10Z,13Z,16Z,19Z))	55.251 (4.352)	19.532 (5.971)	0.0249	0.299	2.83
HMDB08055	PC(18:0/22:5(4Z,7Z,10Z,13Z,16Z))	9.151 (6.281)	3.871 (2.773)	0.0249	0.299	2.36
HMDB06083	Troxerutin	188.555 (18.953)	432.889 (25.759)	0.0250 (W)	0.299	-2.3
HMDB08048	PC(18:0/20:4(5Z,8Z,11Z,14Z))	114.082 (59.935)	56.311 (43.130)	0.0264	0.299	2.03
HMDB00142	Formic acid	4.718 (2.078)	7.489 (3.055)	0.0269	0.299	-1.59
HMDB08057	PC(18:0/22:6(4Z,7Z,10Z,13Z,16Z,19Z))	23.314 (15.829)	11.438 (6.380)	0.0275 (W)	0.299	2.04
HMDB07892	PC(14:0/22:6(4Z,7Z,10Z,13Z,16Z,19Z))	0.405 (0.338)	0.139 (0.090)	0.028	0.299	2.91
HMDB0029205	lysoPC(26:0)	0.227 (0.197)	0.456 (0.235)	0.0293	0.299	-2.01
HMDB07874	PC(14:0/18:2(9Z,12Z))	3.462 (3.478)	0.558 (0.715)	0.0297 (W)	0.299	6.21
HMDB03334	Symmetric dimethylarginine	0.638 (0.399)	1.405 (0.802)	0.0310 (W)	0.299	-2.2
HMDB10394	LysoPC(20:3(8Z,11Z,14Z))	1.213 (0.902)	0.492 (0.500)	0.0310 (W)	0.299	2.46
HMDB08288	PC(20:0/22:6(4Z,7Z,10Z,13Z,16Z,19Z))	0.367 (0.230)	0.186 (0.100)	0.0332	0.299	1.98
HMDB11151	PC(O-16:0/18:2(9Z,12Z))	10.915 (6.853)	5.759 (2.592)	0.0381	0.299	1.9
HMDB13469	SM(d18:0/24:1(15Z)(OH))	1.353 (0.764)	2.168 (1.131)	0.0402 (W)	0.299	-1.6
HMDB13458	PC(o-24:0/18:3(6Z,9Z,12Z))	0.909 (0.441)	0.536 (0.290)	0.0428	0.299	1.7
HMDB08138	PC(18:2(9Z,12Z)/18:2(9Z,12Z))	189.522 (12.500)	60.640 (6.755)	0.0465 (W)	0.299	3.13
HMDB13411	PC(o-16:1(9Z)/16:1(9Z))	0.720 (0.496)	0.362 (0.212)	0.048	0.299	1.99

HMDB07893	PC(14:0/24:0)	2.826 (1.972)	1.441 (0.816)	0.0527	0.299	1.96
HMDB02005	Methionine sulfoxide	2.905 (5.587)	9.258 (7.420)	0.0559 (W)	0.299	-3.19
HMDB01257	Spermidine	26.051 (5.660)	89.579 (6.247)	0.0562 (W)	0.299	-3.44
HMDB07980	PC(16:0/20:3(5Z,8Z,11Z))	114.187 (9.084)	34.137 (4.294)	0.0562 (W)	0.299	3.35
HMDB08046	PC(18:0/20:3(5Z,8Z,11Z))	48.635 (3.365)	12.486 (2.974)	0.0562 (W)	0.299	3.9
HMDB13461	SM(d18:0/26:1(17Z))	1.851 (2.751)	5.516 (3.652)	0.0562 (W)	0.299	-2.98
HMDB13407	PC(o-16:0/20:4(8Z,11Z,14Z,17Z))	16.283 (2.363)	4.541 (6.489)	0.0574 (W)	0.299	3.59
HMDB13408	PC(o-16:0/22:0)	1.202 (0.799)	0.650 (0.357)	0.0584	0.299	1.85
HMDB00177	L-Histidine	117.346 (80.250)	201.700 (10.576)	0.0597	0.299	-1.72
HMDB02095	Malonylcarnitine	0.126 (0.070)	0.278 (0.204)	0.0605	0.299	-2.21
HMDB0013288	Nonanoylcarnitine	0.030 (0.013)	0.020 (0.010)	0.0619	0.299	1.53
HMDB13161	2-Hexenoylcarnitine	0.028 (0.011)	0.036 (0.013)	0.0624 (W)	0.299	-1.29
HMDB00641	L-Glutamine	1282.182 (114.075)	2181.556(112.248)	0.0674 (W)	0.299	-1.7
HMDB08284	PC(20:0/22:2(13Z,16Z))	0.385 (0.283)	0.831 (0.524)	0.0674 (W)	0.299	-2.16
HMDB13437	PC(o-18:2(9Z,12Z)/22:0)	3.332 (2.012)	7.584 (4.483)	0.0674 (W)	0.299	-2.28
HMDB11698	SM(d18:1/26:0)	0.342 (0.384)	0.816 (0.527)	0.0674 (W)	0.299	-2.38
HMDB13420	PC(o-18:0/20:4(8Z,11Z,14Z,17Z))	10.546 (7.165)	4.251 (5.187)	0.0680 (W)	0.299	2.48
HMDB13432	PC(o-18:1(9Z)/20:4(8Z,11Z,14Z,17Z))	13.504 (1.159)	4.365 (6.496)	0.0680 (W)	0.299	3.09
HMDB00064	Creatine	214.718 (5.142)	274.656 (8.021)	0.0682	0.299	-1.28
HMDB08036	PC(18:0/18:0)	3.701 (2.157)	2.135 (1.226)	0.0693	0.299	1.73
HMDB08058	PC(18:0/24:0)	0.513 (0.408)	0.253 (0.148)	0.0714	0.299	2.03
HMDB0029220	lysoPC(26:1(5Z))	0.041 (0.037)	0.074 (0.049)	0.0740 (W)	0.299	-1.84
HMDB10382	LysoPC(16:0)	49.146 (4.941)	20.423 (2.187)	0.0741 (W)	0.299	2.41
HMDB13126	Butenylcarnitine	0.042 (0.026)	0.065 (0.030)	0.0767	0.299	-1.56
HMDB01256	Spermine	13.900 (2.781)	33.411 (2.343)	0.0797 (W)	0.299	-2.4
HMDB10383	LysoPC(16:1(9Z))	2.091 (1.689)	0.868 (0.726)	0.0804 (W)	0.299	2.41
HMDB07973	PC(16:0/18:2(9Z,12Z))	393.386 (3.345)	104.022 (7.478)	0.0804 (W)	0.299	3.78
HMDB07984	PC(16:0/20:5(5Z,8Z,11Z,14Z,17Z))	10.467 (3.902)	2.344 (3.647)	0.0804 (W)	0.299	4.47
HMDB13409	PC(o-16:0/22:6(4Z,7Z,10Z,13Z,16Z,19Z))	5.147 (3.763)	1.855 (2.055)	0.0804 (W)	0.299	2.77
HMDB13422	PC(o-18:0/22:6(4Z,7Z,10Z,13Z,16Z,19Z))	3.178 (2.351)	1.269 (1.459)	0.0804 (W)	0.299	2.5

metabolites MDPI

HMDB00001	1-Methylhistidine	8.982 (5.183)	18.600 (14.227)	0.0837	0.306	-2.07
HMDB01565	Phosphorylcholine	11.500 (6.164)	8.189 (3.228)	0.0952 (W)	0.3203	1.4
HMDB01414	Putrescine	2.854 (4.745)	11.380 (9.753)	0.0952 (W)	0.3203	-3.99
HMDB07883	PC(14:0/20:4(5Z,8Z,11Z,14Z))	1.320 (1.109)	0.353 (0.361)	0.0952 (W)	0.3203	3.74
HMDB13413	PC(o-16:1(9Z)/18:2(9Z,12Z))	6.675 (5.612)	1.625 (2.962)	0.0952 (W)	0.3203	4.11
HMDB13415	PC(o-16:1(9Z)/20:4(8Z,11Z,14Z,17Z))	9.792 (8.000)	2.453 (4.196)	0.0952 (W)	0.3203	3.99
HMDB13442	PC(o-20:0/20:4(8Z,11Z,14Z,17Z))	2.046 (1.189)	1.251 (0.836)	0.1084	0.3466	1.64
HMDB13439	PC(o-20:0/18:3(6Z,9Z,12Z))	6.074 (4.756)	2.217 (2.027)	0.1105 (W)	0.3466	2.74
HMDB00086	Glycerophosphocholine	27.227 (13.633)	51.522 (37.164)	0.1119 (W)	0.3466	-1.89
HMDB13451	PC(o-22:1(13Z)/20:4(8Z,11Z,14Z,17Z))	1.781 (1.227)	0.831 (0.874)	0.1119 (W)	0.3466	2.14
HMDB00211	myo-Inositol	193.609 (61.828)	257.911 (109.714)	0.1153	0.3466	-1.33
HMDB00254	Succinic acid	22.382 (7.859)	28.833 (8.759)	0.1192 (W)	0.3466	-1.29
HMDB00190	L-Lactic acid	728.100 (36.948)	982.600 (32.938)	0.1208	0.3466	-1.35
HMDB0013328	Pimelylcarnitine	0.019 (0.010)	0.019 (0.024)	0.1273 (W)	0.3466	1.01
HMDB13448	PC(o-22:0/20:4(8Z,11Z,14Z,17Z))	0.722 (0.563)	0.278 (0.299)	0.1285 (W)	0.3466	2.6
HMDB0000791	L-Octanoylcarnitine	0.146 (0.070)	0.113 (0.094)	0.1308 (W)	0.3466	1.29
HMDB00510	Aminoadipic acid	5.159 (9.036)	22.489 (29.655)	0.1308 (W)	0.3466	-4.36
HMDB00073	Dopamine	1.882 (2.126)	5.710 (6.195)	0.1308 (W)	0.3466	-3.03
HMDB10384	LysoPC(18:0)	12.381 (9.881)	6.340 (8.196)	0.1308 (W)	0.3466	1.95
HMDB07899	PC(14:1(9Z)/14:0)	2.033 (1.720)	0.454 (0.527)	0.1308 (W)	0.3466	4.48
HMDB07969	PC(16:0/16:1(9Z))	28.981 (19.201)	16.492 (11.209)	0.1308 (W)	0.3466	1.76
HMDB07974	PC(16:0/18:3(6Z,9Z,12Z))	9.956 (8.512)	2.754 (3.977)	0.1308 (W)	0.3466	3.62
HMDB10169	SM(d18:1/16:0)	74.382 (53.457)	32.219 (32.462)	0.1308 (W)	0.3466	2.31
HMDB12108	LysoPC(17:0)	0.772 (0.457)	0.501 (0.442)	0.1383 (W)	0.3619	1.54
HMDB10395	LysoPC(20:4(5Z,8Z,11Z,14Z))	3.283 (1.901)	2.049 (2.151)	0.1487 (W)	0.3619	1.6
HMDB00042	Acetic acid	65.573 (33.076)	47.100 (17.794)	0.1504	0.3619	1.39
HMDB01875	Methanol	1149.391 (133.482)	1940.989 (118.47)	0.1519 (W)	0.3619	-1.69
HMDB00651	Decanoylcarnitine	5.242 (16.802)	0.113 (0.153)	0.1519 (W)	0.3619	46.34
HMDB0029221	lysoPC(28:1(5Z))	0.240 (0.139)	0.185 (0.123)	0.1519 (W)	0.3619	1.3
HMDB07989	PC(16:0/22:5(4Z,7Z,10Z,13Z,16Z))	38.419 (25.506)	17.223 (21.359)	0.1519 (W)	0.3619	2.23

HMDB13429	PC(o-18:1(9Z)/18:2(9Z,12Z))	6.619 (4.826)	2.998 (2.580)	0.1519 (W)	0.3619	2.21
HMDB13444	PC(o-20:1(11Z)/20:4(8Z,11Z,14Z,17Z))	3.932 (2.852)	1.767 (1.975)	0.1519 (W)	0.3619	2.23
HMDB13416	PC(o-16:1(9Z)/22:0)	0.736 (1.316)	2.195 (2.517)	0.1554 (W)	0.3661	-2.98
HMDB13339	3-Hydroxy-11Z-octadecenoylcarnitine	0.017 (0.009)	0.022 (0.008)	0.1577 (W)	0.3673	-1.24
HMDB00187	L-Serine	388.455 (379.978)	601.000 (302.518)	0.1597 (W)	0.368	-1.55
HMDB00112	Gamma-Aminobutyric acid	49.527 (14.636)	62.278 (24.820)	0.1695	0.3863	-1.26
HMDB00159	L-Phenylalanine	151.755 (14.752)	243.067 (17.609)	0.1754 (W)	0.3956	-1.6
HMDB13131	Hydroxyhexanoycarnitine	0.045 (0.022)	0.063 (0.035)	0.1835	0.3993	-1.39
HMDB13133	Methylmalonylcarnitine	0.134 (0.147)	0.261 (0.190)	0.1835 (W)	0.3993	-1.95
HMDB00158	L-Tyrosine	129.073 (8.159)	201.489 (13.247)	0.1835 (W)	0.3993	-1.56
HMDB08287	PC(20:0/22:5(7Z,10Z,13Z,16Z,19Z))	0.312 (0.160)	0.223 (0.121)	0.1846	0.3993	1.4
HMDB06461	Linoelaidyl carnitine	0.247 (0.691)	0.027 (0.016)	0.1960 (W)	0.4121	9.15
HMDB00181	L-Dopa	0.425 (0.592)	0.193 (0.196)	0.1962 (W)	0.4121	2.2
HMDB07876	PC(14:0/18:3(9Z,12Z,15Z))	0.486 (0.335)	0.260 (0.270)	0.1963 (W)	0.4121	1.87
HMDB00593	PC(18:1(9Z)/18:1(9Z))	202.537 (15.932)	79.489 (9.802)	0.2014 (W)	0.4145	2.55
HMDB08059	PC(18:0/24:1(15Z))	0.424 (0.237)	0.709 (0.417)	0.2014 (W)	0.4145	-1.67
HMDB13124	Propenoylcarnitine	0.027 (0.013)	0.035 (0.014)	0.2051	0.4182	-1.29
HMDB02250	Dodecanoylcarnitine	0.063 (0.033)	0.047 (0.038)	0.2098 (W)	0.4197	1.33
HMDB01348	SM(d18:1/18:0)	56.600 (6.735)	106.956 (7.463)	0.2098 (W)	0.4197	-1.89
HMDB13465	SM(d18:0/20:2(11Z,14Z))	0.440 (0.353)	0.155 (0.260)	0.2240 (W)	0.4438	2.85
HMDB00824	Propionylcarnitine	0.317 (0.215)	0.208 (0.261)	0.2299 (W)	0.4471	1.52
HMDB03416	D-Arginine	58.218 (22.602)	52.389 (42.053)	0.2299 (W)	0.4471	1.11
HMDB02014	cis-5-Tetradecenoylcarnitine	0.222 (0.161)	0.131 (0.147)	0.2388 (W)	0.4602	1.69
HMDB13426	PC(o-18:1(9Z)/16:0)	13.220 (4.920)	16.791 (8.196)	0.2429	0.464	-1.27
HMDB00243	Pyruvic acid	5.800 (3.289)	8.322 (6.053)	0.2501	0.4649	-1.43
HMDB01406	Niacinamide	7.555 (2.354)	8.867 (2.593)	0.2514	0.4649	-1.17
HMDB13462	SM(d18:0/14:1(9Z)(OH))	4.056 (3.243)	1.394 (2.304)	0.2543 (W)	0.4649	2.91
HMDB13464	SM(d18:0/16:1(9Z))	12.105 (9.833)	3.335 (5.889)	0.2543 (W)	0.4649	3.63
HMDB08037	PC(18:0/18:1(11Z))	73.809 (8.413)	100.067 (6.481)	0.2579	0.4649	-1.36
HMDB00191	L-Aspartic acid	628.936 (94.124)	1123.044 (68.999)	0.2610 (W)	0.4649	-1.79

HMDB00172	L-Isoleucine	160.755 (113.657)	228.867 (126.489)	0.2610 (W)	0.4649	-1.42
HMDB00562	Creatinine	96.482 (83.054)	152.056 (107.787)	0.2610 (W)	0.4649	-1.58
HMDB08119	PC(18:1(9Z)/22:2(13Z,16Z))	0.611 (0.276)	0.781 (0.393)	0.27	0.477	-1.28
HMDB13463	SM(d18:0/16:1(9Z)(OH))	2.709 (1.305)	2.111 (1.009)	0.2758	0.4831	1.28
HMDB02366	Tiglylcarnitine	0.047 (0.023)	0.058 (0.019)	0.2786	0.4841	-1.22
HMDB00552	3-Methylglutarylcarnitine	0.052 (0.063)	0.086 (0.092)	0.2873 (W)	0.4912	-1.66
HMDB00182	L-Lysine	312.545 (18.585)	371.556 (26.579)	0.2873 (W)	0.4912	-1.19
HMDB08285	PC(20:0/22:4(7Z,10Z,13Z,16Z))	0.290 (0.125)	0.379 (0.232)	0.2899	0.4917	-1.3
HMDB00271	Sarcosine	34.609 (8.866)	52.311 (8.438)	0.2947 (W)	0.492	-1.51
HMDB10386	LysoPC(18:2(9Z,12Z))	12.575 (10.251)	6.376 (9.974)	0.2947 (W)	0.492	1.97
HMDB00895	Acetylcholine	1.636 (0.512)	2.067 (1.012)	0.3036 (W)	0.501	-1.26
HMDB00904	Citrulline	56.055 (7.077)	62.389 (8.531)	0.3049 (W)	0.501	-1.11
HMDB13445	PC(o-22:0/18:3(6Z,9Z,12Z))	1.620 (0.958)	1.240 (0.616)	0.3189	0.5201	1.31
HMDB13410	PC(o-16:1(9Z)/14:1(9Z))	0.058 (0.030)	0.042 (0.027)	0.3230 (W)	0.5225	1.37
HMDB01517	AICAR	0.029 (0.070)	0.039 (0.050)	0.3254 (W)	0.5225	-1.32
HMDB00161	L-Alanine	902.364 (41.072)	1387.556 (73.626)	0.3312 (W)	0.5239	-1.54
HMDB07871	PC(14:0/18:0)	71.573 (8.274)	136.061 (19.163)	0.3312 (W)	0.5239	-1.9
HMDB0029206	lysoPC(28:0)	0.227 (0.120)	0.301 (0.211)	0.3394	0.5294	-1.32
HMDB00033	Carnosine	3.100 (2.605)	3.578 (1.981)	0.3412 (W)	0.5294	-1.15
HMDB00870	Histamine	10.964 (7.733)	6.656 (5.313)	0.3421 (W)	0.5294	1.65
HMDB10405	LysoPC(24:0)	0.141 (0.091)	0.196 (0.168)	0.3567	0.5425	-1.39
HMDB01511	Phosphocreatine	11.591 (5.168)	13.611 (5.557)	0.3616 (W)	0.5425	-1.17
HMDB13334	9,12-Hexadecadienoylcarnitine	0.023 (0.009)	0.027 (0.013)	0.3695	0.5425	-1.2
HMDB00687	L-Leucine	268.109 (29.050)	398.067 (23.609)	0.3702 (W)	0.5425	-1.48
HMDB00696	L-Methionine	99.927 (11.743)	159.222 (12.827)	0.3702 (W)	0.5425	-1.59
HMDB13466	SM(d18:0/22:1(13Z)(OH))	9.338 (5.905)	5.793 (4.892)	0.3702 (W)	0.5425	1.61
HMDB13467	SM(d18:0/22:2(13Z,16Z)(OH))	7.077 (5.076)	4.081 (3.790)	0.3702 (W)	0.5425	1.73
HMDB13125	Hydroxypropionylcarnitine	0.012 (0.009)	0.010 (0.015)	0.3711 (W)	0.5425	1.2
HMDB00157	Hypoxanthine	3.909 (4.497)	3.856 (1.412)	0.4028 (W)	0.5811	1.01
HMDB13418	PC(o-18:0/18:2(9Z,12Z))	11.401 (7.759)	6.168 (5.581)	0.4030 (W)	0.5811	1.85

HMDB13404	PC(o-16:0/16:1(9Z))	3.072 (1.237)	2.597 (1.280)	0.4113	0.5822	1.18
HMDB12107	SM(d18:1/24:1(15Z))	42.082 (18.696)	49.133 (18.635)	0.4117	0.5822	-1.17
HMDB06469	Linoleyl carnitine	0.053 (0.073)	0.040 (0.048)	0.4119 (W)	0.5822	1.35
HMDB13207	9-Hexadecenoylcarnitine	0.038 (0.027)	0.029 (0.016)	0.4247 (W)	0.5962	1.29
HMDB07972	PC(16:0/18:1(9Z))	289.000 (17.154)	350.000 (28.754)	0.435	0.6055	-1.21
HMDB07993	PC(16:0/24:1(15Z))	0.303 (0.162)	0.249 (0.137)	0.4386	0.6055	1.22
HMDB13438	PC(o-18:2(9Z,12Z)/24:0)	0.590 (0.278)	0.490 (0.286)	0.4399	0.6055	1.2
HMDB13341	PC(o-14:0/16:0)	0.273 (0.124)	0.237 (0.070)	0.4523	0.6149	1.15
HMDB13336	3-Hydroxyhexadecanoylcarnitine	0.011 (0.007)	0.008 (0.006)	0.4524	0.6149	1.28
HMDB13331	3, 5-Tetradecadiencarnitine	0.055 (0.037)	0.055 (0.061)	0.4561 (W)	0.6159	-1.01
HMDB00300	Uracil	6.873 (4.451)	5.644 (2.415)	0.4682	0.6259	1.22
HMDB13335	3-Hydroxyhexadecadienoylcarnitine	0.034 (0.019)	0.026 (0.014)	0.4694 (W)	0.6259	1.28
HMDB11697	SM(d18:1/24:0)	14.816 (7.064)	12.699 (6.110)	0.4882	0.6469	1.17
HMDB00194	Anserine	11.655 (4.474)	18.356 (7.697)	0.4940 (W)	0.6469	-1.57
HMDB07869	PC(14:0/16:0)	4.925 (2.785)	5.960 (3.854)	0.4943	0.6469	-1.21
HMDB00201	L-Acetylcarnitine	4.656 (2.803)	4.827 (4.771)	0.5027 (W)	0.6499	-1.04
HMDB00162	L-Proline	321.727 (23.949)	409.444 (28.608)	0.5027 (W)	0.6499	-1.27
HMDB00679	Homocitrulline	12.918 (9.515)	10.889 (1.055)	0.5175 (W)	0.6619	1.19
HMDB13405	PC(o-16:0/18:0)	3.401 (2.897)	4.899 (3.946)	0.5183 (W)	0.6619	-1.44
HMDB00123	Glycine	79.545 (8.566)	91.389 (9.764)	0.5313	0.6745	-1.15
HMDB00050	Adenosine	4.436 (3.476)	5.033 (3.064)	0.5429 (W)	0.6851	-1.13
HMDB00684	L-Kynurenine	2.322 (1.546)	1.923 (1.348)	0.5513	0.6882	1.21
HMDB13431	PC(o-18:1(9Z)/20:1(11Z))	0.526 (0.520)	0.686 (0.660)	0.5518	0.6882	-1.3
HMDB01873	Isobutyric acid	0.491 (0.592)	0.356 (0.167)	0.5841 (W)	0.7204	1.38
HMDB02815	LysoPC(18:1(9Z))	9.400 (5.107)	8.089 (5.441)	0.5859	0.7204	1.16
HMDB08054	PC(18:0/22:4(7Z,10Z,13Z,16Z))	7.413 (2.987)	6.530 (4.210)	0.5903	0.7204	1.14
HMDB13327	Dodecanedioylcarnitine	0.032 (0.011)	0.034 (0.010)	0.5922	0.7204	-1.08
HMDB00883	L-Valine	330.818 (53.744)	390.222 (96.943)	0.5947 (W)	0.7204	-1.18
HMDB13406	PC(o-16:0/20:0)	0.715 (0.355)	0.950 (0.900)	0.6027 (W)	0.726	-1.33
HMDB13402	PC(o-14:0/16:1(9Z))	0.017 (0.029)	0.018 (0.020)	0.6178 (W)	0.7384	-1.07

HMDB00357	3-Hydroxybutyric acid	3.818 (2.814)	4.089 (2.396)	0.6200 (W)	0.7384	-1.07
HMDB00848	Stearoylcarnitine	0.040 (0.018)	0.036 (0.011)	0.642	0.7594	1.09
HMDB00122	D-Glucose	22.773 (65.589)	2.989 (1.450)	0.6483 (W)	0.7594	7.62
HMDB00929	L-Tryptophan	57.400 (16.230)	52.956 (14.983)	0.6484 (W)	0.7594	1.08
HMDB00705	Hexanoylcarnitine	0.053 (0.019)	0.048 (0.030)	0.6532	0.7595	1.1
HMDB05065	Oleoylcarnitine	0.079 (0.054)	0.079 (0.102)	0.6556 (W)	0.7595	-1
HMDB00062	L-Carnitine	15.755 (5.928)	18.411 (9.286)	0.7039 (W)	0.8111	-1.17
HMDB00222	L-Palmitoylcarnitine	0.081 (0.066)	0.061 (0.044)	0.7103 (W)	0.814	1.32
HMDB13128	Valerylcarnitine	0.196 (0.094)	0.247 (0.179)	0.7323 (W)	0.8347	-1.26
HMDB00131	Glycerol	104.136 (47.310)	110.267 (37.863)	0.7568	0.8375	-1.06
HMDB00812	N-Acetyl-L-aspartic acid	145.091 (4.223)	152.044 (8.198)	0.7582	0.8375	-1.05
HMDB12101	SM(d18:1/18:1(9Z))	11.927 (4.843)	12.641 (5.431)	0.7597	0.8375	-1.06
HMDB00034	Adenine	78.409 (9.147)	82.567 (3.845)	0.7607	0.8375	-1.05
HMDB05066	Tetradecanoylcarnitine	0.071 (0.059)	0.049 (0.018)	0.7611 (W)	0.8375	1.46
HMDB00044	Ascorbic acid	14.391 (5.172)	13.456 (8.405)	0.7631	0.8375	1.07
HMDB0059659	Trans-3-hydroxy-L-proline	11.576 (5.477)	10.899 (4.172)	0.7638	0.8375	1.06
HMDB13434	PC(o-18:1(9Z)/24:0)	0.501 (0.223)	0.505 (0.310)	0.7664 (W)	0.8375	-1.01
HMDB00251	Taurine	39.836 (15.435)	41.633 (14.618)	0.7939	0.8631	-1.05
HMDB00134	Fumaric acid	1.664 (0.659)	1.744 (0.875)	0.8163	0.8815	-1.05
HMDB01539	Asymmetric dimethylarginine	0.443 (0.126)	0.425 (0.217)	0.8196	0.8815	1.04
HMDB00097	Choline	19.118 (10.509)	20.189 (10.629)	0.8238 (W)	0.8815	-1.06
HMDB13330	3-Hydroxy-cis-5-tetradecenoylcarnitine	0.017 (0.010)	0.018 (0.008)	0.8274	0.8815	-1.05
HMDB13326	trans-2-Dodecenoylcarnitine	1.881 (0.888)	1.950 (0.701)	0.8519	0.9007	-1.04
HMDB13443	PC(o-20:0/22:0)	0.636 (0.181)	0.619 (0.229)	0.854	0.9007	1.03
HMDB13205	9-Decenoylcarnitine	1.796 (0.843)	1.856 (0.708)	0.8674	0.9104	-1.03
HMDB00125	Glutathione	10.736 (13.737)	7.867 (4.196)	0.8820 (W)	0.9211	1.36
HMDB13129	Glutaconylcarnitine	0.033 (0.026)	0.036 (0.035)	0.9091 (W)	0.9402	-1.09
HMDB01881	Propylene glycol	3.973 (3.547)	3.589 (3.571)	0.9092 (W)	0.9402	1.11
HMDB00863	Isopropyl alcohol	68.427 (223.034)	1.200 (0.490)	0.9390 (W)	0.9589	57.02
HMDB00479	3-Methylhistidine	8.373 (4.916)	8.211 (4.542)	0.9405	0.9589	1.02

HMDB02013	Butyrylcarnitine	0.251 (0.135)	0.405 (0.500)	0.9408 (W)	0.9589	-1.62
HMDB13414	PC(o-16:1(9Z)/20:0)	24.397 (8.810)	24.688 (11.403)	0.9494	0.963	-1.01
HMDB13449	PC(o-22:0/22:3(10Z,13Z,16Z))	0.124 (0.056)	0.125 (0.074)	0.9606	0.9698	-1.01
HMDB00259	Serotonin	7.139 (22.011)	0.514 (0.191)	0.9697 (W)	0.9743	13.89
HMDB00149	Ethanolamine	19.591 (10.959)	19.589 (9.834)	0.9997	0.9997	1

Supplementary Table S1. Metabolite Concentrations (μM) for CP vs Control PM Brain Extracts. Those compounds highlighted in bold are considered statistically, significantly different ($p<0.05$; $q<0.05$). *t*-test values were calculated as a default and values with (W) were calculated using the Wilcoxon Mann Whitney test.

metabolites MDPI

Disease	Age	Sex	PM Delay (hours)
Control	79	M	27
Control	70	M	26
Control	60	M	28
Control	16	F	26
Control	53	M	16
Control	22	M	20
Control	50	M	29
Control	45	M	20
Control	8	M	36
Control	20	F	23
Control	27	M	12
CP	29	F	27
CP	70	M	15
CP	71	M	12
CP	27	M	21
CP	10	M	9
CP	19	F	21
CP	60	M	48
CP	79	M	24
CP	58	F	2

Supplementary Table S2. A list of the available demographic information. PM-post-mortem.
